# Editorial: Familial Cancer in China: From Detection to Screening and Management

**DOI:** 10.3389/fonc.2022.916814

**Published:** 2022-04-29

**Authors:** Tianhui Chen, Ying Yuan

**Affiliations:** ^1^ Department of Cancer Prevention/Zhejiang Cancer Institute, Cancer Hospital of the University of Chinese Academy of Sciences (Zhejiang Cancer Hospital); Institute of Basic Medicine and Cancer (IBMC), Chinese Academy of Sciences, Hangzhou, China; ^2^ Department of Medical Oncology, Key Laboratory of Cancer Prevention and Intervention, Ministry of Education, The Second Affiliated Hospital, Zhejiang University School of Medicine; Cancer Center, Zhejiang University, Hangzhou, China

**Keywords:** familial cancer, family history, cancer screening, Genetic Counseling, Chinese familial cancer

Familial cancer is defined through diagnosis of the same or related cancers in two or more family members. Familial cancer is an important part of cancer genetics, as it can help us better understand cancer etiology. Family history of cancer offers a cheap tool for cancer prevention because healthy family members can be offered guidance about avoiding environmental risk factors and screening options for early detection of tumors. However, in China, research of familial cancer faces many difficulties. At first, though there are numerous clinicians in China, only a few of them are well versed in the diagnosis, treatment and family management of familial cancer. Additionally, obtaining a reliable family history may not be straightforward because, in many local cultures, cancers, particularly advanced cancers, are not openly discussed between family members. Even in medical settings, information to the patients or their family members may be limited.

This Research Topic aims to collect and present the latest data of Chinese familial cancer, including incidence, screening strategies, clinical practice, and family management. We aim to expand the knowledge and understanding of familial cancer in China. In studies reporting familial risks, it is important that the authors consider the reliability of the family histories reported. In this Research Topic, we have received a total of 35 submissions and selected 5 articles (one Corrigendum) contributed by 48 authors, which have received 8,979 views and nearly 1,800 downloads so far. Our collection covers different types of cancer and various study designs. The main reason for the very low acceptance of submissions was due to the recent change of the journal policy “manuscripts consisting solely of bioinformatics, computational analysis, or predictions of public databases which are not accompanied by validation (independent cohort or biological validation *in vitro* or *in vivo*) will not be accepted in any of the sections of Frontiers in Oncology”.

Wen et al. investigated site-specific variation in familial cancer by comparing family history (FH), multiple primary cancer (MPC), age at onset (AO), and male-to-female sex ratio (MFSR) among 8768 patients with esophageal squamous cell carcinoma (ESCC) and gastric cardia adenocarcinoma (GCA). They found the proportion of familial cancer among upper gastrointestinal tract cancer (UGIC) may decrease site-specifically, which may help to set up a better screening strategy or individualized treatments for UGIC patients.

Using a large population-based cohort study-Nutrition Intervention Trial from Linxian, Henan Province, one of the high-incidence regions of upper gastrointestinal (UGI) cancer in China, Yang et al. found that a family history (FH) of UGI cancer in first-degree relatives was associated with an increased risk of esophageal squamous cell carcinoma (ESCC) and gastric cardia carcinoma (GCC) incidence and mortality, but no associations were found with risk of gastric non-cardia carcinoma (GNCC) incidence and mortality. Those data suggest the role of the FH of UGI cancer in the risk of ESCC and GCC incidence and mortality.

Predisposition of germline BRCA1/2 mutations increases the risk of breast and ovarian cancer in females, but the mutation prevalence and spectrum are highly ethnicity-specific with different recurrent mutations being reported in different populations. Li et al. performed hybridization-based target sequencing of BRCA1/2 in 530 Chinese ovarian cancer patients and subsequently conducted haplotype analysis of six short tandem repeat (STR) markers in the patients with recurrent mutations to determine their founder effect. They found 28.3% (150/530) of the ovarian cancer patients in this Henan cohort as BRCA germline mutation carriers (117 in BRCA1 and 34 in BRCA2), including one with concurrent mutations in both BRCA1 and BRCA2. Haplotype analysis revealed a region of 0.6 MB genomic length spanning BRCA1 highly conserved across all the independent carriers of BRCA1:c.5470_5477del, supporting it as a founder mutation in Henan population. Nevertheless, a larger cohort consisting samples from nationwide multi-centers is also warranted to confirm this finding, which may help us to understand the founder effect of BRCA mutations in Chinese and design a cost-effective screening test for the high-risk population. Additionally, retrospective analysis in a subgroup of serous ovarian cancer patients revealed BRCA germline mutation status was not associated with the progression-free survival (PFS), while a nuclear expression of Ki-67 over 50% of the malignant cells appeared to be an independent predictor for a shorter PFS.

Shen et al. summarize the susceptibility genes and genetic syndromes associated with familial breast cancer (FBC) by searching the PubMed database for related articles published between January 2000 and August 2021.They identified 16 FBC-related genes and divided them into three types (high-, medium-, and low-penetrance) of genes according to their relative risk ratios, and summarized the currently available screening strategies for FBC and discussed those available for high-risk Chinese populations. Early detection of FBC is pivotal for the improvement of 5-year survival. For the Chinese population, different screening strategies need to be adopted based on unique genetic information, while genetic counseling and genetic testing shall consider family history, mutant genes, and genetic syndromes.

The study by Bao et al. identified five significant clusters from 14 cervical cancer screening strategies in terms of accuracy, cost, and efficiency using hierarchical clustering methods. The data suggest that hierarchical clustering methods offer an alternative way to synthetically assess screening strategies based on multiple indicator systems. The cluster including primary HPV screening with genotyping and cytology triage showed an optimal balance among benefit, cost, and efficiency, providing clinical and methodological evidence on the choice of HPV-based screening strategies, which may help health decision-makers choose an appropriately high-performance strategy for local cervical cancer prevention and the affordability of health resources.

## Author Contributions

TC drafted the manuscript. TC and YY revised the manuscript and all authors approved the submission.

## Funding

This work was supported by grants from Key Research-Development Program of Zhejiang Province (2017C03013), Sino-German Mobility Programme (M-0008), National Key Research-Development Program of China (2019YFE0198800 and 2021YFC2500400), Ten-Thousand Talents Plan of Zhejiang Province (2021R52020), and Startup Funds for Recruited Talents at Zhejiang Cancer Hospital.

## Conflict of Interest

The authors declare that the research was conducted in the absence of any commercial or financial relationships that could be construed as a potential conflict of interest.

## Publisher’s Note

All claims expressed in this article are solely those of the authors and do not necessarily represent those of their affiliated organizations, or those of the publisher, the editors and the reviewers. Any product that may be evaluated in this article, or claim that may be made by its manufacturer, is not guaranteed or endorsed by the publisher.

